# A nationwide survey of first aid training and encounters in Norway

**DOI:** 10.1186/s12873-017-0116-7

**Published:** 2017-02-23

**Authors:** Håkon Kvåle Bakke, Tine Steinvik, Johan Angell, Torben Wisborg

**Affiliations:** 1 Mo i Rana Hospital, Helgeland Hospital Trust, Mo i Rana, Norway; 2 Anaesthesia and Critical Care Research Group, Faculty of Health Sciences, IKM, University of Tromsø, 9037 Tromsø, Norway; 3 Department of Anaesthesiology and Intensive Care, University Hospital of North Norway, Tromsø, Norway; 4 Lawyers Leiros & Olsen AS, Tromsø, Norway; 5 Faculty of Law, University of Tromsø, Tromsø, Norway; 6 Hammerfest Hospital, Department of Anaesthesiology and Intensive Care, Finnmark Health Trust, Hammerfest, Norway; 7 Norwegian National Advisory Unit on Trauma, Division of Emergencies and Critical Care, Oslo University Hospital, Oslo, Norway

**Keywords:** First aid, Laypersons, Legislation, Education, Training, Cardiac arrest, Trauma

## Abstract

**Background:**

Bystander first aid can improve survival following out-of-hospital cardiac arrest or trauma. Thus, providing first aid education to laypersons may lead to better outcomes. In this study, we aimed to establish the prevalence and distribution of first aid training in the populace, how often first aid skills are needed, and self-reported helping behaviour.

**Methods:**

We conducted a telephone survey of 1000 respondents who were representative of the Norwegian population. Respondents were asked where and when they had first aid training, if they had ever encountered situations where first aid was necessary, and stratified by occupation. First aid included cardio-pulmonary resuscitation (CPR) and basic life support (BLS). To test theoretical first aid knowledge, respondents were subjected to two hypothetical first aid scenarios.

**Results:**

Among the respondents, 90% had received first aid training, and 54% had undergone first aid training within the last 5 years. The workplace was the most common source of first aid training. Of the 43% who had been in a situation requiring first aid, 89% had provided first aid in that situation. There were considerable variations among different occupations in first aid training, and exposure to situations requiring first aid. Theoretical first aid knowledge was not as good as expected in light of the high share who had first aid training. In the presented scenarios 42% of respondent would initiate CPR in an unconscious patient not breathing normally, and 46% would provide an open airway to an unconscious road traffic victim. First aid training was correlated with better theoretical knowledge, but time since first aid training was not.

**Conclusions:**

A high proportion of the Norwegian population had first aid training, and interviewees reported high willingness to provide first aid. Theoretical first aid knowledge was worse than expected. While first aid is part of national school curriculum, few have listed school as the source for their first aid training.

**Electronic supplementary material:**

The online version of this article (doi:10.1186/s12873-017-0116-7) contains supplementary material, which is available to authorized users.

## Background

In instances of out-of-hospital cardiac arrest (OHCA) and trauma, survival may rely on swift and correct first aid from bystanders. Cardio-pulmonary resuscitation (CPR) after OHCA can more than double the survival rate, and can improve chances of complete neurological recovery [1]. Few studies have investigated first aid in trauma, but it is estimated that 1.8–5% of trauma deaths could be prevented if bystanders provided airway clearance and bleeding control [2]. In Norway, 54–76% of OHCA victims receive bystander CPR, and 62–81% of trauma patients receive basic life support (BLS) from bystanders [3–6]. These rates of bystander first aid coverage are at the high end of the reported worldwide range from 15 to 55% [7, 8].

Bystanders with first aid training are more likely to provide first aid, and such training is associated with better first aid quality [4, 9, 10]. Thus, provision of first aid education to laypersons is considered an important means of improving OHCA and trauma outcomes [2, 11–13]. Since it may not be of benefit or feasible to train the entire population in first aid, such training should target high-risk groups and schoolchildren [14]. To guide first aid education efforts, it is of interest to investigate the extent of first aid training in the populace.

The present study aimed to establish the prevalence and distribution of first aid training in the populace, how often first aid skills are needed, and self-reported helping behaviour.

## Methods

### The Study area

Norway, at the time of the survey had 5.1 million inhabitants, and an area of 323 772 km^2^. First aid training is part of the national school curriculum for grades 7 and 10. Since 2003 first aid training has also been compulsory for obtaining a drivers license. Employees in schools and kindergartens, and fishermen are required by law to be able to perform first aid. All other occupational groups are unregulated by Norwegian law, and only subject to their various occupational standards, if any. First aid guidelines are provided by the Norwegian First Aid Council, a cooperative body comprising non-government organisations and government agencies with focus on first aid training, and the Norwegian Resuscitation Council. There are no statutory requirements for first aid training providers to follow these guidelines or other standards.

### Survey

In April 2014 a telephone survey was conducted by Norstat telemarketing company (Oslo, Norway). The study was to include 1000 interviewees to attain a first aid training rate within +/- 3% of the true value of the population. Participants were 15 years of age and older, and all interviews were conducted in Norwegian. Census data from the Norwegian Bureau of Statistics indicated that our sample was proportional to the Norwegian populace composition with regards to gender and county of residence. A digital dialler system randomly selected telephone numbers (30% to landline telephones, 70% to mobile phones) within each county until the sample was completed. The participants could respond to the survey upon contact, or arrange to be interviewed at a later time.

The questionnaire used in the telephone survey was developed by the authors in cooperation with Norstat. The participants were asked 13 questions, including gender, age, county of residence, level of education and income. There were also a total of 12 follow-up questions. A translated version of the questions can be found in Additional file 1. In two of the questions hypothetical scenarios of first aid were presented, scenario 1 concerned non-arrest trauma, and scenario 2 concerned CPR. The participants were asked what they would do in those situations and allowed to speak freely, while the interviewer noted if the participant mentioned any of the listed measures (Table 2). The interviewer marked on a checklist what answers were given without prompting or suggesting measures. The term “first aid” did in this setting comprise both resuscitative, and non-resuscitative, basic, or advanced life support.

For the scenarios a total score was computed. For scenario 1, calling the emergency telephone number, providing an open airway (either opening the airway, checking for breathing, or placing patient in recovery position), control of bleeding, and keeping the patient warm were each given 1 point for a correct answer. For scenario 2, calling the emergency telephone number and initiating CPR were each given 1 point for a correct answer. The scores were then added up.

### Statistical analysis

Statistical analyses were performed using SPSS Statistics for Mac version 21 (IBM Corp., Armonk, NY, USA). The tests used to examine correlations are specified in the Results section. For logistic regressions, we performed preliminary analyses for normality, linearity, and homoscedasticity. Several occupations had 100% first aid training, and when controlling for occupation these occupations were exempt from analysis. Correlations were determined for hypothesis-generating purposes and were not corrected for multiple comparisons.

### Ethics and consent

The study was reviewed by the Regional Committee for Medical and Health Research Ethics, University of Tromsø (ref. 2014/8/REK nord). The need for ethical approval was waived due to the study design. All participants were informed of the study’s purpose.

## Results

**Fig. 1:**
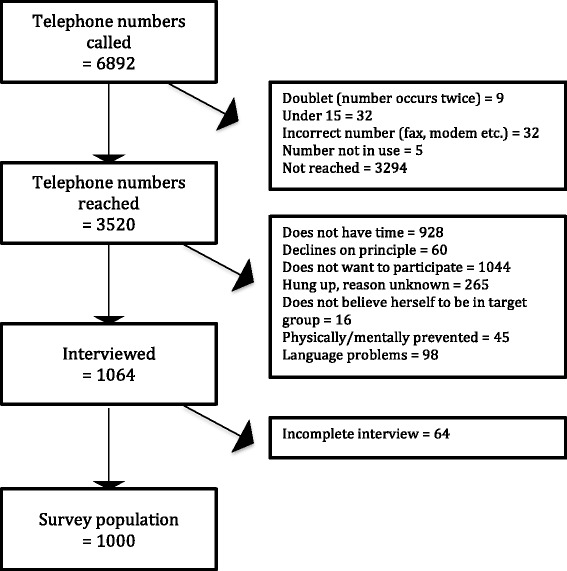
Flow diagram illustrating the dial process for the telephone survey

We called a total of 6892 telephone numbers, reaching 3520 individuals, and successfully interviewing 1000 persons (28% of those reached and eligible) (see Fig. 1). Median age was 45 (Range 15–99, Inter-quartile range (IQR): 30), 500/1000 were women. Among these 1000 interviewees, 426 (43%) had been present for a situation requiring first aid, of whom 378 (89%) provided first aid, 46 (11%) had not provided first aid, and 2 (0.5%) could not remember whether they had provided first aid or not. The main reason given for not contributing was that other bystanders intervened (35/46). Persons with first aid training were more likely to have been in a situation requiring first aid compared to those that did not have first aid training (*p* < 0.001; logistic regression; controlling for age, gender, and occupation), but not more likely to intervene (overall *p* = 0.254, chi-square test, or when controlled for age, and gender *p* = 0.514, logistic regression).

Of the 1000 interviewees, 895 (90%) had received first aid training. The median number of trainings was 3 (range, 0–300; IQR, 73). The median time since last training was 4 years (range, 0–64; IQR, 10), and 54% (540) had received first aid training within the last 5 years. Based on the mean for the last 10 years, the annual training rate was 6.1%. Interviewees with first aid training were younger (*t*-test, mean 44 vs 56 years, *p* < 0.01), and men were more likely than women to be trained first aiders (91% trained vs 85% trained, chi-square test, *p* = 0.02). The interviewees sources of first aid training are given in Table 1. There were significant differences in age distribution for the different sources of training (Comparison of means, one-way ANOVA, *p* < 0.01), with those stating “School” or “Part of occupational training” as source for first aid training comparatively younger than other groups (post hoc Bonferroni).

**Table 1:** Sources of first aid training for 895 interviewees with first aid training (more than one source possible per person)

Source of first aid training	Percent	*Number*
Work	52%	465/895
Non-governmental organisation	21%	184/895
Military service	16%	138/895
Driver’s licence training	13%	118/895
School	13%	112/895
Association/club/organisation	6%	50/895
Part of occupational training	5%	44/895
Other	6%	54/895

**Fig. 2:**
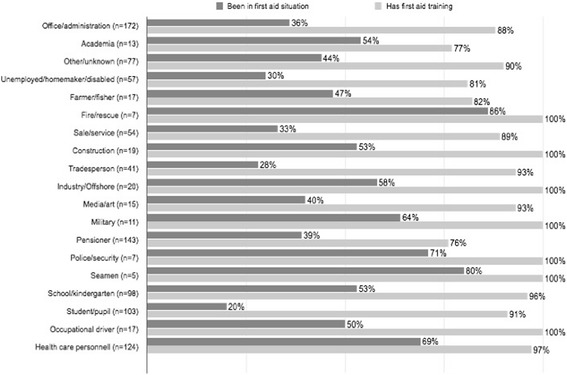
The proportion with first aid training and the proportion that have encountered situations where provision of first aid was necessary, by different occupations

Occupation was correlated with differences in exposure to situations requiring first aid (*p* < 0.001, chi-square test), and in first aid training (*p* < 0.001, chi-square test). Figure 2 presents data regarding occupational exposure and first aid training. Gender was correlated with differences in occupation (*p* < 0.001, chi-square test), with a higher percentage of men in occupations where 100% of employers had first aid training than in occupations where less than 100% had first aid training (91% vs 46% men, chi-square test, *p* < 0.01)

Among the 895 interviewees with first aid training, 160 had been in a situation requiring first aid since their last first aid course, of whom 124 (78%) felt that their first aid training had adequately prepared them for the first aid situation. Neither the time since last training nor number of trainings affected the likelihood of feeling prepared (*p* = 0.360 and *p* = 0.11, logistic regression). Of the 1000 interviewees, 715 (72%) felt that they would know what to do in a situation requiring first aid, while 68 (7%) did not think they could physically perform first aid. A minority, 122 (12%) expressed reservations, and were unwilling to perform certain first aid measures, which 31% of the 122 explained as being related to fear of infection. The proportion with reservations did not vary between those trained in first aid or not (*p* =0.58, chi-square test).

The respondents were presented with two first aid scenarios. The answers are given in Table 2. First aid course correlated with higher score (Score 0–5, *p* < 0.001, B = 0.378, Multiple linear correlation, adjusted for gender, age, and whether respondent was health care personnel). Time since last first aid course was not associated with difference in first aid score when adjusted for age, gender, number of first aid courses and whether respondent was health care personnel (*p* = 0.2, Multiple linear regression). Whereas number of first aid courses taken was associated with a slightly higher score (*p* = 0.39, B = 0.082, Multiple linear regression)

**Table 2:** Respondents’ answers to two scenarios presented as part of telephone survey

First aid measure	Percent	Number
You happen to pass by a road traffic accident where a car has driven off the road, and the driver is sitting unconscious in the front seat. What do you do?		
Secure scene	28%	279/1000
Call emergency dispatch	82%	819/1000
Open airway	43%	433/1000
Check breathing	36%	364/1000
Place in recovery position	16%	156/1000
Check for/stop bleeding	11%	110/1000
Keep patient warm	16%	156/1000
Check for pulse	31%	311/1000
You happen to pass by a man who is lying unconscious. He does not breathe normally, but emits short gasps. What do you do?		
Call emergency dispatch	64%	644/1000
Start CPR	42%	424/1000
Open airway ^a^	29%	286/1000
Place in recovery position ^a^	33%	326/1000
Check for pulse	18%	176/1000

^a^ without starting CPR

## Discussion

This study reports a high rate of exposure (43%) to situations requiring first aid actions, and a remarkably high willingness to act amongst the informants (89%). Our results revealed that 90% of the participants had at least some first aid training, with 54% having had a first aid course within the last 5 years. The workplace was the most common source of first aid training, with considerable variation in first aid training among different occupations. Although first aid is part of the school curriculum, only 13% listed school as a source for their first aid training. Willingness to perform first aid was high. Of the 43% who had been in a situation requiring first aid, 89% had provided first aid.

The present proportion of persons with first aid training was high compared to reports from Australia, New Zealand, USA, and Sweden that show proportions of 45–79% [15–19]. Our yearly overall training rate, at 6% of the population per year, was also high compared to a recent American study [20]. We found that 54% of interviewees had received training within the last 5 years, which is higher than the rates of 25–37% in international literature [15, 17, 19]. Most prior studies have only examined CPR training and cannot be directly compared. However most of the Norwegian First Aid Council’s courses are centred on CPR, and; thus, most of the respondents with first aid training likely had CPR training. It would be of interest for future studies to further investigate the content (CPR, treatment of injuries, other conditions, and their relative proportions), mode (classic instruction, video-based, or other), and lengths of first aid training offered to the populace.

As in earlier studies, we found that older persons were less likely to be trained in first aid [15, 16]. While prior studies have reported no gender differences or that women are more likely to have first aid training, here we found that men were more likely to be trained [15, 16]. However, this correlation was weak, and the gender discrepancy was likely related to differences in occupation. Occupation also showed only a weak correlation with first aid training but the occupation groups were composite, which may have weakened correlations with particular professions.

Most respondents were willing to perform first aid in an appropriate situation, and 89% of those who had been in such a situation had provided assistance. Moreover, most of those who did not provide assistance refrained from doing so because the patients were receiving first aid from someone else on scene. In Norway, a high percentage of patients receive bystander first aid, both for out-of-hospital cardiac arrest (54–76%) and trauma (62–81%) [3–6]. Interestingly, we found no correlation between first aid training and having provided bystander first aid. Thus, the high proportion of patients receiving first aid may be related to willingness to help more than the rate of first aid training. However, our study was not designed to answer that question. Other studies show that bystanders providing first aid are more likely to have first aid training [9]. Future studies should endeavour to establish whether such behaviour is indeed tied to first aid training, or rather to confounders, such as occupations.

Though most respondents had first aid training the answers to the theoretical scenarios were not as uplifting. Only 42% suggested to start CPR in a scenario where a person was found unconscious and not breathing normally, and 43% suggested to provide an open airway to an unconscious road traffic victim. Two Norwegian surveys have found similar rates for first aid knowledge, though they were carried out by organisations selling first aid courses, and not published in peer-review journals [21]. Previous first aid training was associated with a higher total score in the scenarios. Number of courses taken also, but to a lesser extent, whereas. Time since last course was not associated with a higher score, if analysis was adjusted for number of courses taken. This finding should be interpreted with care though, as theoretical knowledge not necessarily reflects practical first aid skills.

The Norwegian school system includes first aid as part of the national curriculum, as recommended by the American Heart Association [22, 23].

However, the curriculum does not specify what first aid measures pupils should master, how often and at what levels first aid training should be given, or what courses or guidelines should be followed [23]. This is left to the discretion of the individual teacher and school. Only 13% of interviewees listed school as a source for their first aid training, which was markedly lower than in a similar study from Sweden [16]. This percentage seems rather low considering that first aid is part of the curriculum at nationally mandatory levels. Given that those who listed school as a source for first aid training were younger this finding may possibly be due to recall bias. Teaching first aid in school can potentially reach the vast majority of the population, and evidence suggests that first aid can be taught from a young age [11, 24, 25]. Future studies should examine what and how much first aid training is actually performed in the school system, and whether there exists any barriers to such training.

Our present study had several limitations, such as the low number of respondents willing to answer the survey (28%), which was somewhat lower than in previous studies. There is likely a selection bias where people who are not trained in first aid are less likely to participate in the study. This is a major limitation and may have had an impact on the results of the survey. Additionally, several survey questions were subject to recall bias. The survey was conducted in Norwegian only, likely excluding members of minority groups. As shown in a Swedish study, minorities are less likely to have first aid training [16]. Furthermore, the survey did not distinguish between CPR and non-CPR first aid, or between those who had first aid training before being in a situation requiring first aid and those who received training afterwards. In the scenario questions used to measure first aid knowledge, measures that appear early in the ABCDE mnemonic seemingly received better scores. This could mean respondents scored worse in some measures because they were not prompted to go on. Furthermore the respondents may have made different assumptions of the situation described in the scenario questions as they were presented with a rather short, and oral description of the scene.

## Conclusions

A high proportion of the Norwegian population is exposed to situations requiring first aid actions, and the large majority is willing to act. Many have received first aid training, with more than half trained within the last 5 years. Willingness to provide first aid is high. While first aid is part of the school curriculum, few interviewees listed school as their source of first aid training. The study identifies several areas for future studies, including the mode and content of first aid training; and how first aid is actually taught in the school system.

## Additional file

Additional file 1: English translation of questionnaire (translated from Norwegian). (DOCX 19 kb)

## Abbreviations

ABCDE: Airways, breathing, circulation, disability, exposure/environment
BLS: Basic life support
CPR: Cardiopulmonary resuscitation
IQR: Inter-quartile range
OHCA: Out of hospital cardiac arrest
