# Supplementary material for: A nationwide survey of first aid training and encounters in Norway
Source: BMC Emerg Med. 2017 Feb 23;17:6. doi: 10.1186/s12873-017-0116-7 (PMC5322636; doi:10.1186/s12873-017-0116-7)
Supplement: Additional file 1: — English translation of questionnaire (translated from Norwegian). (DOCX 19 kb) [file 12873_2017_116_MOESM1_ESM.docx]

**Additional file 1**

English translation of questionnaire (translated from Norwegian)

Question A

L,Gender

1. Male

2. Female

Question B

What is your age?

Two digits possible

Question C

What is your county of residence?

1. Finnmark
2. Troms
3. Nordland
4. Nord-Trøndelag
5. Sør-Trøndelag
6. Møre og Romsdal
7. Sogn og Fjordane
8. Hordaland
9. Rogaland
10. Vest-Agder
11. Aust-Agder
12. Telemark
13. Vestfold
14. Buskerud
15. Oppland
16. Hedmark
17. Østfold
18. Akershus
19. Oslo

Question 1

What is your occupation?

Open comment

Question 2

Have you ever been in a situation where first aid skills were required?
1. Yes

2. No

3. Don't know/Don't remember

Question 2b

If 1 in Q2

Did you yourself provide assistance in that situation?

1. Yes

2. No

3. Don't remember

Question 3a

If 1 in Q2b

What was your main reason for doing so?

Open comment

Question 3b

If 2 in Q2b

What was the most important reason that you did not?

1. Others intervened

2. Was afraid of inflicting injury/do something wrong

3. Was uncertain of what I should do

4. I found myself unable to act

5. Other - open comment

Question 4

Have you ever had a first aid course, or received first aid training?

1. Yes

2. No

3. Don't know

Question 5

If 1 in Q4

Where did you receive this course or receive first aid training

1. NGO (Red Cross, Norwegian People's aid etc)

2. Through work

3. Military service

4. Part of obtaining a driver's license

5. Other - open comment

Multiple answers possible

Question 6

If 1 in Q4

How many times have you participated in a first aid course/training?

Question 7

If 1 in Q4

When was the last time (which year) you received first aid training?

Question 8

If 1 in Q4

Have you, after you, at a later point, been in a situation where first aid skills were required?

1. Yes

2. No

3. Don't know

Question 9

If 1 in Q8

Did you find that your first aid training had made you adequately prepared for that situation?

1. Yes

2. No

3.Don't know

Question 10

If2 in Q8

What was lacking? - Open comment

Question 11

Today, do you feel that you would know what to do in the event of sudden illness (such as a heart attack or sudden loss of consciousness) or an if accident should occur?

1. Yes

2. No

3. Don't know

Question 12

If 2 in Q11

Why not? - Open comment

Question 13

Would you be able to perform first aid if sudden illness (such as a heart attack or sudden loss of consciousness) or an accident should occur?

1. Yes

2. No

3. Don't know

Question 14

If 2 in Q13

Why not? - Open comment

Question 15

Are there any particular first aid measures you would not be willing to do?

1. Yes

2. No

3. Don't know

Question 16

If 1 in Q15

What would you not be willing to do, and why? - Open comment

Question 17

I will now present two different scenarios

Number 1: You happen to come across a traffic accident where a car has driven off the road, and the driver is sitting unconscious in the front seat. What do you do?

(Respondent answers freely and interviewer notes if any of the following are mentioned:)

1. Secure the scene

2. Call 113 (emergency phone number)/ambulance

3. Open airway

4. Check for breathing

5. Check for pulse

6.Check for/stop bleeding

7. Keep patient warm

8. Stabilise neck

9. Put patient in recovery position

10. Start CPR

11. Other, -what (Interviewer notes)

Question 18

You happen to pass by a man who is lying unconscious. He does not breathe normally, but emits short gasps. What do you do?

(Respondent answers freely and interviewer notes if any of the following are mentioned:)

1. Call 113 (emergency phone number)/ambulance

2. CPR/Mouth to mouth/Chest compressions

3. Open airway

4. Check for pulse

5. Put patient in recovery position

6. Other, -what (Interviewer notes)

Question 19

What is your highest completed level of education?

1. Primary school

2. Secondary school (1-3 years after primary school)

3. University/College (1-3 years after secondary school)

4. University/College (4 years or more after secondary school)

5. Unanswered

Question 20

What is your household's total gross annual income?

1. Less than 100 000 NOK
2. 100-200 000 NOK
3. 201-300 000 NOK
4. 301-400 000 NOK
5. 401-500 000 NOK
6. 501-600 000 NOK
7. 601-700 000 NOK
8. 701-800 000 NOK
9. 801-900 000 NOK
10. 901-1 000 000 NOK
11. 1.001-1 100 000 NOK
12. 1.101-1 200 000 NOK
13. 1.201-1 300 000 NOK
14. 1.301-1 400 000 NOK
15. 1.401-1 500 000 NOK
16. Above 1 500 000 NOK
17. Do not wish to answer
18. Don't know
